# Colored Tattoo Ink Screening Method with Optical Tissue Phantoms and Raman Spectroscopy

**DOI:** 10.3390/ma14123147

**Published:** 2021-06-08

**Authors:** Filip Sadura, Maciej S. Wróbel, Katarzyna Karpienko

**Affiliations:** Department of Metrology and Optoelectronics, Faculty of Electronics, Telecommunications and Informatics, Gdańsk University of Technology, G. Narutowicza 11/12, 80-233 Gdańsk, Poland; filip.sadura@pg.edu.pl (F.S.); maciej.wrobel@pg.edu.pl (M.S.W.)

**Keywords:** Raman spectroscopy, tattoo inks, optical tissue phantoms

## Abstract

Due to the increasing popularity of tattoos among the general population, to ensure their safety and quality, there is a need to develop reliable and rapid methods for the analysis of the composition of tattoo inks, both in the ink itself and in already existing tattoos. This paper presents the possibility of using Raman spectroscopy to examine tattoo inks in biological materials. We have developed optical tissue phantoms mimicking the optical scattering coefficient typical for human dermis as a substitute for an in vivo study. The material employed herein allows for mimicking the tattoo-making procedure. We investigated the effect of the scattering coefficient of the matrix in which the ink is located, as well as its chemical compositions on the spectra. Raman surface line scanning has been carried out for each ink in the skin phantom to establish the spatial gradient of ink concentration distribution. This ensures the ability to detect miniature concentrations for a tattoo margin assessment. An analysis and comparison of the spectra of the inks and the tattooed inks in the phantoms are presented. We recommend the utilization of Raman spectroscopy as a screening method to enforce the tattoo ink safety legislations as well as an early medical diagnostic screening tool.

## 1. Introduction

Tattoos have become one of the most popular forms of body modification and people, especially young ones, are no longer perceiving them as a controversial element of their image [1]. The popularity of tattoos is growing, as can be confirmed by surveys conducted in the United States, where, in 2017, as many as 29% of adults said they had at least one tattoo [2]. That is up from 20% in 2015 and 13% in 2007 [3]. Tattoos are made by the injection of a pigment into the body during a series of skin punctures with a needle containing the ink. As a result, the ink reaches the dermis layer, embedding the pigment, and thus, changing the skin color in the desired pattern [4]. Professional tattoos require specialized equipment, like an electric-powered tattoo machine, which allows for making hundreds of punctures per minute [5], multi-colored inks, which are available in hundreds of shades and colors [6], and antiseptics. Tattoo ink producers, to meet the customers’ expectations, provide inks in various embodiments, such as vegan inks and UV-fluorescent inks [7]. The latter might be used in research for the accurate identification of biopsy sites [8]. Besides allergies, tattoos can also cause other health problems. The ink injected into the body can trigger an immune over-response, while small particles of the pigment migrate to the lymph nodes and liver [9]. As a rare consequence, unnecessary surgical treatment can be undertaken due to the misidentification of melanoma metastases in the lymph nodes due to the presence of ink in the organs [10]. There are no regulations for tattoo inks, as they are for cosmetics or medical products. Tattoo inks, especially for cosmetics tattoos, may contain mixtures of unknown pigments and other substances potentially dangerous to human health [11,12]. The Council of Europe, in a resolution from 2008, recommends two analytical methods for the analysis and detection of hazardous chemical compounds in the composition of tattoo inks: gas and liquid chromatography with mass spectrometry (GC/LC-MS) [13]. However, the applicability of those methods is limited since they require advanced instrumentation, trained personnel, and specific protocols. Sample handling and preparation protocols are available for cosmetics and food but not for tattoo inks. Such methods may be applied to samples of inks, while measurements of tattoos are possible only for biopsy samples, so there must be a premise, such as a severe reaction, warranting the biopsy procedure. Those methods are unavailable for in vivo measurements to ascertain the ink safety, for example, during the screening tests.

The composition of tattoo inks is a mixture of pigments [10], solvents, and supplementary substances that improve the applicative properties of the ink, like viscosity or drying [14,15]. The most important component in the ink is the pigment, responsible for the ink’s color. Pigments are crystalline particles, often with low water solubility, that remain in a solid state even after injection into living tissue, making them difficult to remove [16]. The pigments are marked with the identifier “C.I.” by the International Color Index, according to their chemical structure [17]. Other utilized components are alcohols, such as glycerin, ethanol, isopropyl alcohol, or benzyl alcohol, which stabilize the dispersion of pigments. In addition, the inks contain numerous preservatives that improve the effectiveness of applying the ink into the skin or accelerate wound healing [14] but may also contain contaminants and hazardous chemicals [18]. The procedure carries a risk of the introduction of hazardous, harmful, allergenic, or cancerogenic compounds into the skin. The growing popularity of tattoos and the lack of regulations on the safety of their composition is an alarming factor in public health. This indicates the need for better tools that would allow for ascertaining compliance with regulations should they inevitably come. Especially in the case of already existing tattoos, non-invasive in vivo measuring methods would allow for pre-emptive screening for hazardous substances in the individuals’ tattoos.

As GC/LC-MS methods require a sampling of the inks, they cannot be used to measure an existing tattoo in the skin. A non-invasive optical method, like Raman spectroscopy, can be used to measure the skin in vivo. The Raman spectrum is dependent on a specific chemical structure and is often referred to as the "molecular fingerprint" of a substance, allowing qualitative measurements for chemical identification. It is used, for example, in archeology, to determine the details of the carbon-based black ink composition found in mummies’ bodies [19], or in medicine to detect the components of ink causing an allergic reaction [19]. It is also possible to perform quantitative measurements, as the signal intensity depends on the concentration of the substance. Additionally, for the purpose of tattoos, Raman spectroscopy has been used very scarcely, often with the use of very expensive spectrometers with microscope-based systems [20].

However, the assessment of tattoo composition is complicated due to the matrix they reside in, that is, the skin, because of the measurement noise and variability of biological substances [21]. In order to establish Raman spectroscopy as a viable measurement method for this cause, it is necessary to build spectral databases, calibration models, or machine learning methods [22]. These require the acquisition of the spectra of substances in the designated matrix, which in this case, makes it near impossible to obtain since carcinogenic or otherwise harmful ink contaminants would have to be introduced into human skin. This cannot be ethically accomplished in vivo, while ex vivo skin samples are tough to obtain and their parameters change very rapidly over time. This raises the necessity of a stable and reliable material with modifiable properties as a substitute for the skin [23,24]. Optical tissue phantoms often serve such a purpose in numerous optical methods. In the case of Raman spectroscopy, the phantoms have been used rarely due to changes in their chemical composition and the target material [25]. We propose that this may not necessarily be a major obstacle since the main phantom Raman bands are outside the most important regions for the investigated molecules.

In this paper, we present the possibility of using Raman spectroscopy to detect tattoo inks in biological materials. We determine the characteristic bands of the Raman spectra of tattoo inks. In addition, we developed phantoms for a Raman spectroscopy study of tattoo inks as a substitute for an in vivo study. The material employed herein allows for mimicking the tattoo-making procedure as it normally would be done by the artist’s hand. We used a tattooed porcine skin as a control. We investigated the effect of the scattering coefficient of the matrix in which the ink is located. We performed Raman surface scanning for each ink in the skin phantoms in order to investigate the spatial gradients of the ink concentration distribution in such a medium.

## 2. Materials and Methods

### 2.1. Raman Spectroscopy

**Figure 1:**
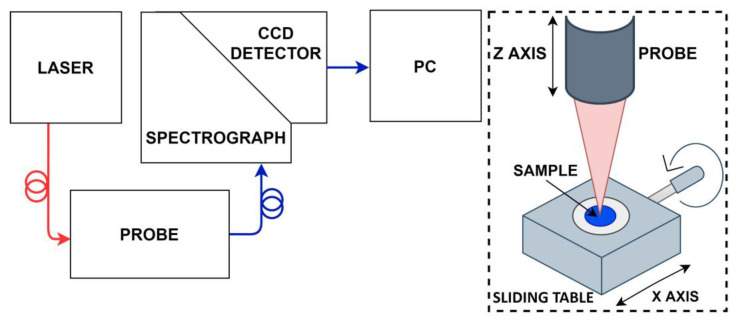
The scheme of the Raman measurement system. The block diagram of the system is shown on the left and the probe scheme and the sample holder on the right.

To measure the Raman spectra, we used a system containing a CCD camera (iDus DU401ABR–DD, Andor Technology Ltd., Belfast, UK), a spectrograph, and an excitation laser at 830 nm wavelength (I0830MU0500MF, Innovative Photonic Solutions, Monmouth Junction, NJ, USA). Figure 1 presents the scheme of the system. The system includes an axis-transmission spectrograph with a diffraction grating optimized for recording the Raman spectrum in the range of about 200–1900 cm^−1^, that is about 835–1000 nm [26]. The system is also equipped with a bundle-to-line fiber-optic probe. The excitation laser is guided through the additional FC/PC threaded single-mode 125 µm excitation fiber. The detection fibers bundle is arranged in a circular pattern at the head of the probe. At the end of the probe, there is a chamber that connects the detection fiber and the excitation fiber. To eliminate laser radiation in the receiving path, the probe has built-in bandpass filters. The lens at the end of the probe allows for gathering the signal from a working distance of about 2.54 cm (2 in) without contact, a safe distance from the sample or patient. The laser spot size is about 0.15 mm. The probe is mounted on a stand that moves along the Z-axis (up–down), which enables setting the laser focus points on the sample. The sample is placed on a sliding table with uniaxial translation and a micrometric screw, which allows for scanning along the horizontal X-axis.

### 2.2. Optical Tissue Phantoms

For the measurements to verify the applicability of Raman spectroscopy for the detection of tattoo inks in biological materials, human tissues were not used in the research due to ethical and practical reasons. The optical tissue phantoms are oftentimes used as a substitute for the tissues since they can mimic precisely specific properties of the tissues [27], in this case, the reduced scattering coefficient. The phantoms were used as a substitute for human dermis. Following the procedure described in the article by [22], three phantoms were prepared with different proportions of glycerin, and thus, with different reduced scattering coefficients. The parameters of the phantoms are shown in Table 1.

**Figure 2:**
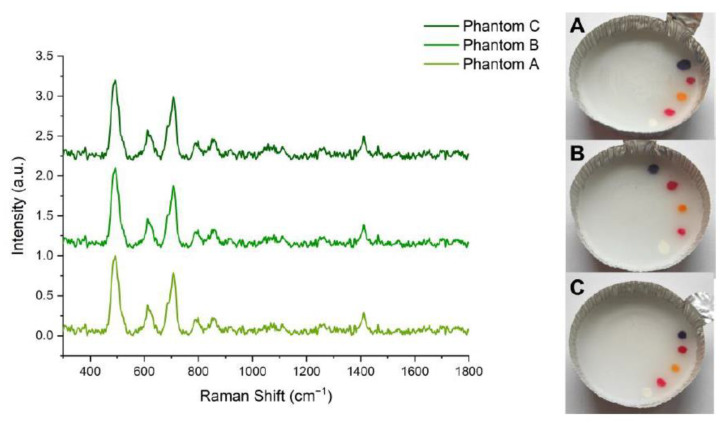
Normalized and baseline-corrected Raman spectra of pure Phantoms A, B, and C (**on left**). Dots tattooed with Inks 1–5 on Phantoms A, B, and C (**on right**).

For the phantom creation, the PDMS and the curing agent were always added in the ratio of 1:10 parts per volume. The phantom components, glycerin and polydimethylsiloxane (PDMS), were thoroughly mixed in the proportions according to Table 1. The resulting mixture was placed in a vacuum chamber for an hour in order to remove air bubbles from the solution. Then, the mixtures were poured into aluminum weigh dishes and put into the oven. They were kept at 60 °C until solidified. Then, after cooling to room temperature, the phantoms were tattooed. The phantoms were tattooed with each of the tested inks, using an electric-powered tattoo machine and 6 mm needles. The needles were introduced in the phantom to a depth of about 3 mm, where the ink was deposited. This procedure was carried out several times to achieve homogeneous dots, the same way normal tattoos are prepared. Approximately 7.83 mg of ink were deposited per 1 cm^2^, which is a typical amount of ink used in a regular tattooing procedure [28]. Using the system described in Section 2.1, the spectra of all the phantoms were measured. Figure 2 shows the tattooed Phantoms A–C and the normalized and baseline-corrected Raman spectra of the pure phantoms.

Due to the different chemical properties of the matrix, the phantoms’ spectra differ from the spectrum of the skin or other tissues but they allow for imitating their optical properties, such as scattering, in the range typical of human skin tissues. The spectra of the phantoms show the bands characteristic for PDMS and glycerin. With an increase in the ratio of glycerin concentration to PDMS, which is responsible for the increase in the reduced scattering coefficient μ_s_′, a slight increase in the intensity of the bands at 1049, 1311, and 1464 cm^−1^, referring to the glycerin, is visible, while the signal intensity for the bands at 492 and 707 cm^−1^, referring to the PDMS, remains unchanged.

### 2.3. Tattoo Inks

There were 5 inks differing in pigment, and thus, color, that were analyzed in this study. We selected the inks from reputable manufacturers who provided the complete product documentation. The composition of the inks was confirmed by Safety Data Sheets and test results from accredited laboratories. The chosen colors were white, red, pink, purple, and orange. The inks’ compositions and descriptions are presented in Table 2.

The main differences in the compositions of the inks used are the pigments. In Ink 1, it is C.I.77891 (titanium oxide), in Ink 2, it is C.I.12475 (Naphthol Red), Ink 3 contains C.I.77891 and C.I.12477, Ink 4 contains a mixture of C.I.77891, C.I.12466, C.I.74160 (Copper (II) Phthalocyanine), and C.I.12475, and in the composition of Ink 5, there is C.I.77891, C.I.21160, and C.I.21108. Besides pigments, the main ingredients in the selected tattoo inks are mostly water, glycerin, isopropyl alcohol, and Hamamelis Virginiana extract.

**Figure 3:**
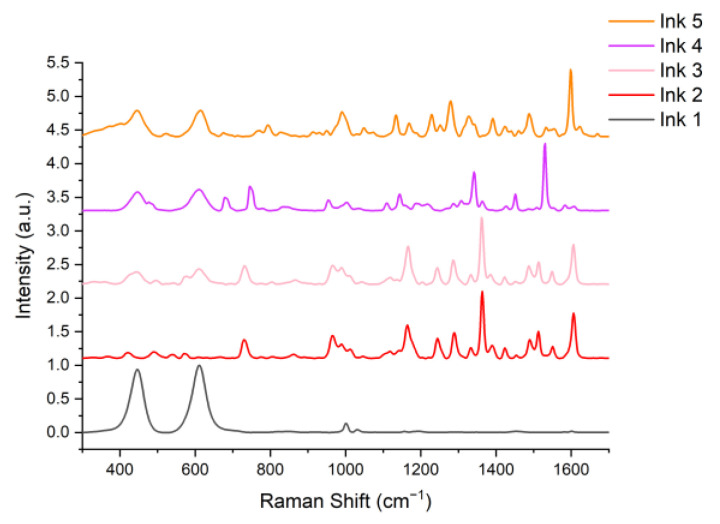
Pre-processed Raman spectra of Inks 1–5.

The inks were characterized prior to their further use in the study. A drop of each ink was measured using the system described in Section 2.1. The results were pre-processed, smoothed with the Savitzky–Golay (SG) method, baseline–corrected, and normalized. The spectra were plotted in the range of 300–1700 cm^−1^ and are presented in Figure 3.

In Ink 1 (white), the color is caused by titanium oxide, characterized by bands at 450 and 610 cm^−1^. These bands are also visible in the spectra of Inks 2, 4, and 5, where titanium oxide was used to obtain brighter colors by enhancing the phenomenon of light scattering (pink, bright orange). Due to the presence of pigments with a similar chemical structure in Inks 2 and 3 (C.I.12477, C.I.12475), their spectra have a similar shape but their characteristic bands are shifted relative to each other: 862, 1047, 1142, and 1289 cm^−1^ for Ink 2, and 668, 1043, 1138, and 1285 cm^−1^ for Ink 3 [29,30]. Inks 2 and 4 share the same pigment (C.I.12475), and thus, these bands can also be seen in Ink 4 yet at a much lower intensity. This pigment is responsible for red, which is a primary color in Ink 2, while it only gives a secondary color impression in Ink 4 (violet). In most of the spectra, one can see the bands characteristic of additives such as glycerin (1040 and 1490 cm^−1^), isopropanol (1450 cm^−1^), and benzyl alcohol, (800 and 1000 cm^−1^). A table listing the characteristic bands of Inks 1–5 can be found in the Supplementary Materials. The table in the Supplementary Materials (SM) provides the association between the found Raman bands of inks with their compositions. The characteristic bands of pigments were detected and compared with available data from the literature. The analysis of these bands confirmed that the detected pigments in the inks are in agreement with manufacturers’ information provided in the datasheets.

## 3. Results and Discussion

**Figure 4:**
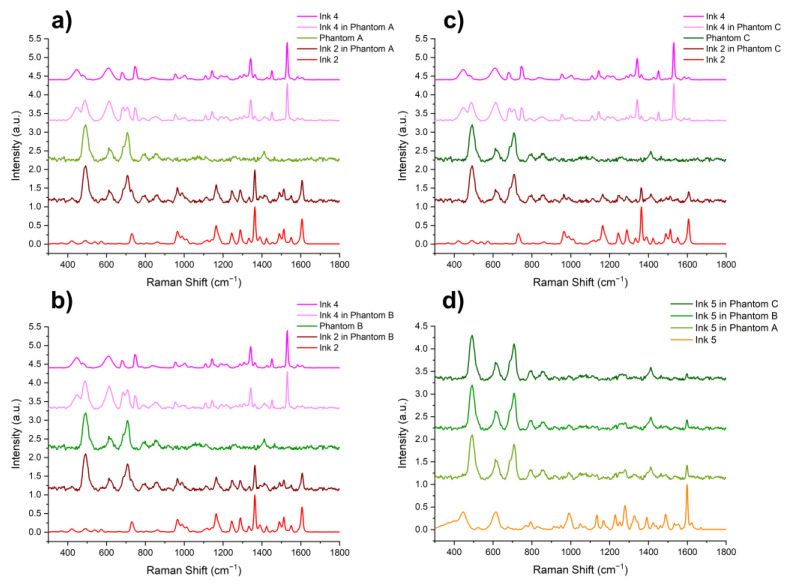
(**a**–**c**) Smoothed and baseline-corrected spectra of Phantoms A, B, and C in comparison to the spectra of tattoos made in them with Inks 2 and 4; (**d**) Ink 5 in comparison to Ink 5 injected into Phantoms A, B, and C.

Using the system described in Section 2.1, the Raman spectra of Inks 1–5 tattooed in Phantoms A–C were measured. The single spectra were measured with an exposure time of 2–5 s, depending on the fluorescence intensity. Then, the results were smoothed using the Savitzky–Golay filter with a 7-point window and a 2nd degree polynomial. Baseline subtraction was used to remove the influence of fluorescence and allow for assessing only the effect of the phantom composition on the resulting spectra. Figure 4a–c presents the spectra of Phantoms A, B, and C, and the spectra of the tattoos made in them with Inks 2 and 4. Figure 4d presents, collectively, the spectra of Ink 5, and Ink 5 injected into Phantoms A, B, and C. This comparison was made to determine the effect of the scattering coefficient of the matrix into which the ink was injected on the spectrum of the tattoo.

As can be seen in Figure 4a–c, the tattoo spectra consist of combinations of bands characteristic of pure ink and the phantom. The bands characteristic of Ink 2, at 1362 and 1606 cm^−1^, are potentially visible in the spectra of tattoos made in all three Phantoms. This also applies to the bands at 1340 and 1530 cm^−1^ corresponding to Ink 4. An increase in the value of the scattering coefficient affects a decrease in the signal intensity of the bands characteristic of the inks. This tendency is especially visible in Figure 4d.

**Figure 5:**
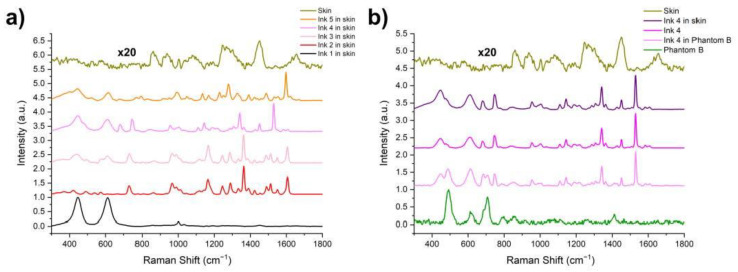
(**a**) Pre-processed Raman spectra of Inks 1–5 tattooed in porcine skin and the spectrum of pure porcine skin. (**b**) Comparison between the pure porcine skin and pure Phantom B spectra, as well as comparison between Ink 4 in Phantom B, reference spectrum of Ink 4, and Ink 4 in the porcine skin.

As a control experiment, we used porcine skin, which was tattooed with the same inks as the phantoms. The spectra of the pure porcine skin and all the inks in the skin were measured. The data is shown in Figure 5. In Figure 5a, we can see the comparison between the pure porcine skin and Inks 1–5 tattooed in the porcine skin. The influence of the skin is hardly visible, as it is overwhelmed by the signal from the inks. A comparison between the skin and the phantom is shown in Figure 5b. The phantoms show only a few prominent peaks, most of which are contained in the lower wavenumber region, below 800 cm^−1^, while most of the inks and skin bands are at the higher wavenumbers, above 800–1000 cm^−1^. The rapid decline of the ex vivo skin is the main reason for its substitution by the phantom materials. The lowering concentration of the inks occurs when the tattoo requires lighter coloring or the shading of the pattern. In such cases, the influence of the ink on the spectra is much lower. This may be very important for tattoo margin delineation. Therefore, we assessed these changes of intensity of the inks in the tattoos.

**Figure 6:**
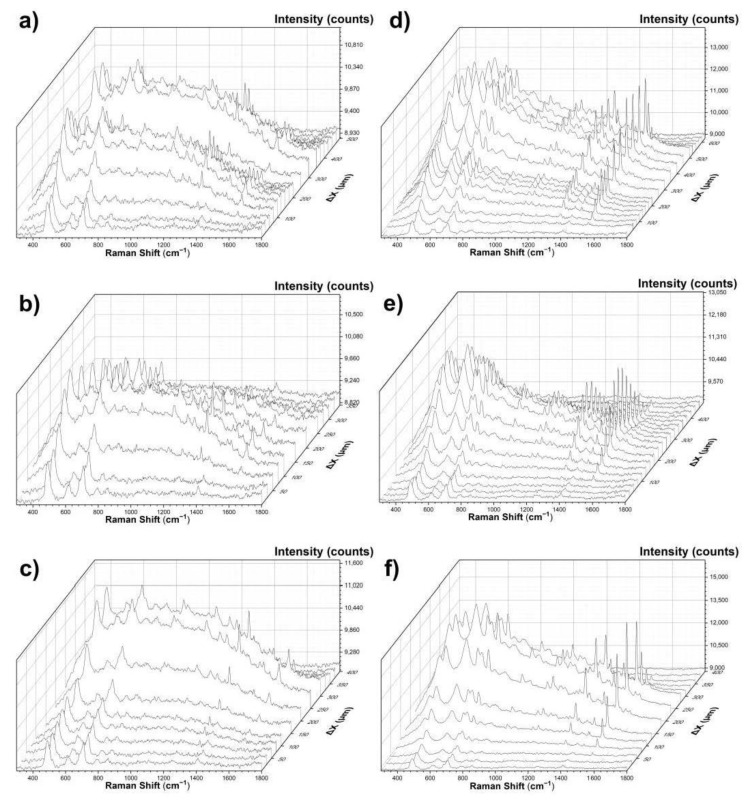
Raman spectra (**a**–**c**) show surface scanning results for Ink 2, respectively, in Phantoms A, B, and C, while the spectra (**d**–**f**) present scanning results for Ink 4 in the same phantoms.

The ink rarely decomposes perfectly even in a biological sample, both horizontally and in-depth. Therefore, scanning of the prepared tattoos was carried out to evaluate the application of Raman spectroscopy for different positions and concentrations of the ink in the tattoo. Raman line scans for Inks 1–5 in each phantom were recorded using the sliding table described in the Section 2.1. Measurements were carried out by moving step-by-step through a circular tattoo along its center (or the chord, for larger phantoms), so that the first and the last spectrum registered an almost pure phantom spectrum, while the intermediate ones are in the middle of the tattoo, thus creating a gradient. The measurements were taken at 10 s of exposure time. The results for the tattoos made with Inks 2 and 4 in Phantoms A, B, and C are shown in Figure 6a–f. The results for the remaining inks are presented in the Supplementary Materials.

**Figure 7:**
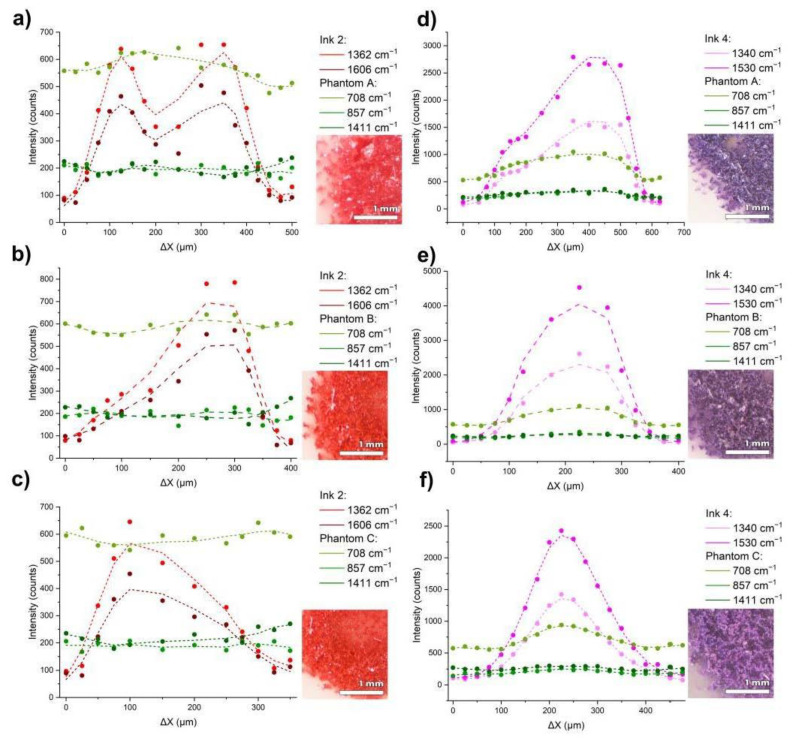
The intensity of the characteristic bands for tattoos made with Ink 2 in Phantoms A, B, and C (**a**–**c**), tattoos made with Ink 4 in Phantoms A, B, and C (**d**–**f**), and phantoms themselves in the offset function of the measurement point. The insets present magnified microscopic images of the tattooed dots.

Along with moving the measuring point further into the tattoo, we can observe changes in the signal intensity. The change in signal intensity gives information about the location of the tattoo and the density of the ink in the matrix. The greatest increase in the signal intensity is observed for the bands characteristic of the inks, such as at 1362 and 1606 cm^−1^ for Ink 2, and 1340, and 1530 cm^−1^ for Ink 4. The increase of the bands characteristic of the phantoms, such as at 708, 857, and 1411 cm^−1^, results mostly from the fluorescence, which enhances the signal almost uniformly in the 500–1700 cm^−1^ range of the spectrum. Figure 7 presents this dependence as a spatial gradient of the ink concentration distribution in the phantom, which was determined by comparing the changes in the intensity of the bands assigned to the inks and the phantoms.

It can be observed that the signal intensity for the bands relative to the inks increases significantly with the density of the ink in the matrix, while for the bands corresponding to the phantoms, such an increase is not observed. Slight changes, especially in the middle, are due to optical background remains, such as fluorescence from the inks, overlapping the signal. In some cases, mainly for Ink 2 in Phantom A, the signal drops in relation to the neighboring positions as a result of the inhomogeneity of the sample surface, which is a consequence of uneven needle punctures, visible in the photos of the phantoms under magnification. Decreasing intensities of inks’ main peaks can be seen, for instance, in the drop of 1530 cm^−1^ band for Ink 4 from near 5000 signal counts to below 2500 counts, along with the increase in the scattering of the phantoms’ material. At the same time, the phantom bands remain mostly unaffected by the amount of scattering or the position on the phantoms. The same phantom band intensity is recorded from outside the area of the tattoo to its center.

## 4. Conclusions

Health concerns regarding the compositions of tattoo inks are gaining awareness by consumers and legislators alike. Following the European Union (EU) Commission report and EU recommendations for the safety of tattoos, new regulations are likely to follow stemming from these guidelines. Thus, new diagnostic tools for regulatory bodies are needed to enable tattoo ink composition control, as well as for screening existing tattoos for the risk of containing harmful substances by physicians. Raman spectroscopy is proposed as a method for the verification of tattoo ink and tattoo composition in vivo.

For this purpose, the viability of optical tissue phantoms was evaluated as tattooed skin substitutes, which can be used as reference standard samples for device calibration and even the establishment of the calibration models themselves. We have presented a range of tattooed optical tissue phantoms with varying scattering coefficients in the typical range for human skin. We compared the created phantoms with a porcine skin model as a control experiment. The phantoms, despite having different chemical compositions from actual skin, are viable due to their reliability and stability of parameters over time. The measurement challenges of optical methods, like the proposed Raman spectroscopy, can be successfully mimicked by varying the phantoms compositions to achieve the desired scattering, and thus, the differences in recorded intensities.

We have detected and identified the pigments in all the inks as specified by the manufacturer. Their characteristic Raman bands were found and are presented in a table in the Supplementary Materials. Of note, the spatial gradient distribution of the inks can indicate their margins should a need arise for localized treatment or removal. Likewise, lighter shades of tattoos can be effectively measured by this technique, as indicated by the measurements of low concentrations of the inks at the edges of the tattoos.

We conclude the utilization of Raman spectroscopy as a viable method of tattoo composition assessment regarding the ink pigments. Along with the use of PDMS-based optical tissue phantoms, which proved to be a valid substitute for human skin, even for such a chemically sensitive method, Raman spectroscopy can be readily available as a screening method for authoritative bodies to enforce the safety legislations and, likewise, as an early medical diagnostic screening tool.

## Figures and Tables

**Table 1:** Compositions and descriptions of the prepared phantoms.

Label	Phantom A	Phantom B	Phantom C
Proportions of glycerin: PDMS vol.:vol. (mL)	1.5:11	2:11	2.5:11
Reduced scattering coefficient (µ_s_’ cm^−1^)	1.25	1.5	1.75

**Table 2:** Compositions and descriptions of inks used in the experiment.

Label (Color)	Ink 1 (White)	Ink 2 (Red)	Ink 3 (Pink)	Ink 4 (Violet)	Ink 5 (Orange)
Name (producer)	White House (World Famous Tattoo INK)	Paul Rogers Red (World Famous Tattoo INK)	Hot Pink (Eternal)	Forbidden City (World Famous Tattoo INK)	Bright Orange (Eternal)
Ingredients	Pigment: C.I.77891, Glycerin, Isopropyl alcohol, Rosin, Hamamelis Virginiana, benzyl alcohol	Water, pigment: C.I.12475, Glycerin, Isopropyl alcohol Rosin, Hamamelis Virginiana, benzyl alcohol	Water, pigment: C.I.77891, C.I.12477, Glycerin, Isopropyl alcohol	Water, pigment: C.I.77891, C.I.12466, C.I.74160, C.I.12475, Glycerin, Isopropyl alcohol, Rosin, Hamamelis Virginiana, DMDM Hydantoin	Water, pigment: C.I.77891, C.I.21160, C.I.21108, Glycerin, Isopropyl alcohol

## Data Availability

Data sharing is not applicable to this article.

## Supplementary Materials

The following are available online at https://doi.org/10.6084/m9.figshare.14562126.v3, Table S1: Characteristic Raman bands for Inks 1–5; Figure S1: Raman spectra (a–c) show scanning results for Ink 1, respectively, in Phantoms A, B, and C, while spectra (d–f) present the scanning results for Ink 3 in the same phantoms; Figure S2: Raman spectra (a–c) show the scanning results for Ink 5, respectively, in Phantoms A, B, and C.
